# Continuity of care and mortality in people with schizophrenia

**DOI:** 10.1192/bjo.2021.965

**Published:** 2021-07-09

**Authors:** Alastair Macdonald, Dimitrios Adamis, Matthew Broadbent, Tom Craig, Rob Stewart, Robin M. Murray

**Affiliations:** National Institute for Health Research (NIHR) Biomedical Research Centre at South London and Maudsley NHS Foundation Trust and King's College London, UK; St. Columba's Hospital, Eire; NIHR Maudsley Biomedical Research Centre, UK; (Emeritus) Institute of Psychiatry, Psychology and Neuroscience, King's College London, UK; Institute of Psychiatry, Psychology and Neuroscience, King's College London, UK; Institute of Psychiatry, Psychology and Neuroscience, King's College London, UK

**Keywords:** Mortality, schizophrenia, deliberate self-harm, outcome studies, suicide

## Abstract

**Background:**

People with schizophrenia have shortened lives. This excess mortality seems to be related to physical health conditions that may be amenable to better primary and secondary prevention. Better continuity of care may enhance such interventions as well as help prevent death by self-injury.

**Aims:**

We set out to examine the relationship between the continuity of care of patients with schizophrenia, their mortality and cause of death.

**Method:**

Pseudoanonymised community data from 5551 people with schizophrenia presenting over 11 years were examined for changes in continuity of care using the numbers of community teams caring for them and the Modified Modified Continuity Index. These and demographic variables were related to death certifications of physical illness from the Office of National Statistics and mortal self-injury from clinical data. Data were analysed using generalised estimating equations.

**Results:**

We found no independent relationship between levels of continuity of care and overall mortality. However, lower levels of relationship continuity were significantly and independently related to death by self-injury.

**Conclusions:**

We found no evidence that continuity of care is important in the prevention of physical causes of death in schizophrenia. However, there is evidence that declining relationship continuity of care has an independent effect on deaths as a result of self-injury. We suggest that there should be more attention focused on the improvement of continuity of care for these patients.

## Background

It has been known for at least 50 years that people with schizophrenia have shortened lives.^1^ Suicide and other violent deaths do not alone explain this. Indeed, Laursen et al^2^ suggest that the difference in suicide rates between people with schizophrenia and the general population is narrowing. But death rates from physical illnesses such as cardiovascular and cerebrovascular disorder remain high relative to the general population.^3^ This may be related to higher incidence in people with schizophrenia. However, for cancer, Toender et al^4^ found that the incidence was actually lower than in controls although mortality was higher. Similarly, although having the same incidence rate as in the general population, survival after a myocardial infarct was lower in people with schizophrenia.^5^ Brink et al^6^ showed markedly lower detection and diagnosis rates of fatal conditions in people with schizophrenia prior to death. Taken together, these data suggest that both primary prevention (better management of cardiovascular and cerebrovascular risk factors such as smoking and obesity^7^) and secondary prevention (better detection and management of treatable conditions already occurring) are needed.

## Continuity of care

Continuity of care may be important in the primary and secondary prevention of physical illness in this group. Hoertel et al^8^ found a relationship between lower continuity and mortality in a sample of 14 515 psychiatric out-patients, especially in those with major psychiatric disorders including schizophrenia.

## Assessing deaths with formal suicide verdicts versus all deaths by self-injury

Lower continuity of care is related to suicide in mood disorders^9^ so may also contribute to suicide in schizophrenia. When in crisis people with schizophrenia experiencing lower continuity of care are significantly less able to identify where to turn.^10^ However, investigation of this outcome may be hampered by changes in the way formal suicide verdicts are reached.^11,12^ Doubts about the validity of suicide verdicts suggest we now investigate all deaths as a result of self-injury, whether or not a formal suicide verdict was reached.

## Aims

We have reported a relationship over 11 years between declining continuity of care – in both relationship and team – and worse clinical outcomes in secondary care patients with schizophrenia.^13^ Here we investigate whether this decline in relationship and team continuity is associated with increased mortality. We also examine this factor in both deaths formally identified as suicide and also in those as a result of other self-injury in schizophrenia.

## Method

Details of the sampling are described in Macdonald et al.^13^ Electronic health record data from the South London & Maudsley NHS Foundation Trust are pseudoanonymised into the Clinical Record Interactive Search System (CRIS).^14^ From CRIS we identified 5552 people with schizophrenia with at least one episode of care in the community presenting between 2006 and 2016. The service has no fixed service duration for patients. These were patients in whom at least 75% of any of the diagnoses ever allocated to them were in either sections F20 or F22 of the ICD-10.^15^

They were examined for changes in ‘team’ continuity of care using the numbers of distinct community teams caring for them. In the electronic care record used in the Trust all active patients outside in-patient care are allocated at least one code indicating a community team. Patients can be removed from one team and added to another, or can be managed by more than one team (‘co-working referral’). In either case the number of teams involved in a patient's care in any 1 year would increase.

All in-patient units are identified; in this study any non-in-patient team was regarded as a community team. All contacts with staff are recorded as connected with a team. Relationship continuity with individual staff members using the Modified Modified Continuity Index (MMCI)^16^ was calculated for all contacts with a community team in the year. This is given by:
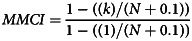


where *k* is the number of different staff seen and *N* is the total number of contacts with all staff in the year; results range from 0 if all staff were seen only once each to 1 if only one staff member was seen throughout. The MMCI is one of several measures of relationship continuity but to our knowledge the only one to have yet been used in secondary mental healthcare patients with schizophrenia^13^

The Index of Multiple Deprivation (IMD)^17^ for the first address each year was obtained via patient Lower Layer Super Output Area codes.

Changes in the Health of the Nation Outcomes Scales (HoNOS) scores^18^ were also obtained, as some of these reflect physical morbidity and are predictive of mortality in this group.^19^ These are 12 five-point scales covering symptoms, functioning, relationships and circumstances completed by clinicians at the start, during and at the end of each episode of care. The circumstances of death of those patients under the care of the Trust when they died were obtained from CRIS event records. Those whose death was because of self-injury were manually identified by one of us (A.M.). Data on smoking history and comorbid diagnoses of substance misuse (‘dual diagnosis’) were extracted from CRIS.

Data linkage between CRIS and Hospital Episode Statistics was used to identify admissions to non-psychiatric hospitals as an indicator of physical morbidity, as were dates and cause of death from certifications from the Office of National Statistics (ONS) via National Health Service (NHS) numbers through an anonymised process. This included formal suicide verdicts, independent of the manual assessment of death because of self-injury directly from CRIS.

Death was related to the continuity of care variables with demographic variables, HoNOS scores, dual diagnosis, smoking history and non-psychiatric hospital admissions as covariates. Deaths because of self-injury were compared with all other causes of death using the same variables.

All ICD-10 codes for underlying causes of death were allocated a flag according to whether or not they were listed under the ONS category ‘treatable’ (in 2016 these were termed ‘Amenable to healthcare interventions’: Office of National Statistics).^20^ These are deaths that, according to the ONS, can be mainly avoided through timely and effective healthcare interventions, including secondary prevention and treatment. No formal suicides, or any causes of death in patients over 74 years old, are regarded by ONS as ‘treatable’.

Each calendar year of the study saw patients enter as they were referred for the first time since the study began, and, during a subsequent year, leave the study either because they were finally discharged, or because they died. The main comparison was between patients who died before final discharge in the 11 years compared with those who remained alive at least until final discharge within the 11 years, taking into account the autocorrelation in the data

### Data analysis

Data were extracted from CRIS using Microsoft SQL Server 2008 R2,^21^ prepared using Visual Foxpro v9.0^22^ and analysed with IBM SPSS v23.^23^ IMD was treated as continuous. The generalised estimating equations (GEE) method was used to analyse longitudinal data. Little's Missing Completely at Random (MCAR) test was used to examine any systematic missing values. For GEE analysis an exchangeable working correlation matrix structure was assumed, with link function identity.

The main GEE model was constructed with the mortality variable (dead/alive) as the dependent variable, and MMCI and number of teams caring for the patient as the independent variables of interest, to which were added as covariates gender, age, ethnicity, main diagnosis (F20 or F22), diagnosis of mental and behavioural disorders because of psychoactive substance use (F10–F19), smoking habits, HoNOS, IMD, the number of discharges from non-psychiatric hospital and the calendar year from 2006 to 2016.

The final most parsimonious model was conducted by dropping, one by one, the non-significant variables guided by the Corrected Quasi Likelihood under Independence Model Criterion (lower values – better fit). Another GEE model was constructed using the same variables but including individual HoNOS scale scores. Finally, further models were constructed using deaths from conditions deemed ‘treatable’ and deaths because of self-injury versus all other causes of death.

### Ethics of research

All procedures contributing to this work comply with the ethical standards of the relevant national and institutional committees on human experimentation and with the Helsinki Declaration of 1975, as revised in 2008. CRIS was approved as a data-set for secondary analysis by Oxfordshire Research Ethics Committee C, reference 08/H0606/71. The use of data in this project was approved by the CRIS oversight committee ref 16–103.

## Results

The derivation of the sample has already been reported.^13^ One patient was removed from analysis as some of their event data was from another patient. In total, 5551 patients contributed 36 264 patient-years between 2006 and 2016. There were 4298 patients (89.4% of those with data on smoking) who had any record of current or past smoking; 511 (10.6%) had never smoked. In 742 (13.4% of the total sample) the smoking record was missing.

There were 487 (8.8%) patients who had at some point an additional diagnosis of mental and behavioural disorders because of psychoactive substance use (F10–F19).

In total, 1042 patients (18.8% of the whole sample) died during the study period of 11 years: 144 (2.6%) died more than a year after final discharge. We found that 898 (16.2%) died while active patients. Details of the sample are shown in Table 1, which also describes those who died from illnesses or other ‘natural causes’ and those who died after self-injury, whether or not their deaths were certificated as suicides.

**Table 1:** Description of sample

Status at final discharge from Trust, death or end of study	*n* (%)	Male, *n* (%)	Diagnosis predominately F20 (%)	White ethnic group, *n* (% of non-missing ethnicity)	Age at discharge, death or end of study: mean (s.d.)	Last MMCI before discharge, death or end of study, mean (s.d.)	Last IMD before discharge, death or end of study, mean (s.d.)	Last number of teams before discharge, death or end of study, mean (s.d.)	Last total HoNOS before discharge, death or end of study, mean (s.d.)
Alive	4653 (83.8)	2968 (63.8)	3895 (83.7)	1771 (38.7)	50.2 (15.3)	0.72 (0.27)	32.5 (10.6)	1.20 (0.62)	9.76 (5.3)
Death by ‘natural causes’	858 (15.5)	472 (55.0)	695 (81.0)	493 (58.6)	67.1 (15.1)	0.79 (0.28)	31.2 (11.4)	1.17 (0.57)	11.40 (5.2)
Death by self-injury	40 (0.7)	32 (80.0)	30 (75.0)	15 (37.5)	40.3 (12.5)	0.71 (0.27)	32.5 (11.0)	1.40 (0.84)	9.61 (5.0)
Total	5551 (100)	3472 (62.5)	4620 (83.2)	2279 (41.7)	52.7 (16.5)	0.73 (0.19)	32.3 (10.7)	1.20 (0.61)	10.00 (5.4)

MMCI, Modified Modified Continuity Index; IMD, Index of Multiple Deprivation; HoNOS, Health of the Nation Outcomes Scales.

### Longitudinal analysis of data

Little's MCAR test (χ^2^ = 130.731, d.f. = 6511, *P* = 1.0) showed that the missing data appeared to be missing completely at random.

The final most parsimonious GEE model on the associations with mortality is presented in Table 2.

**Table 2:** Parameter estimates of the final generalised estimating equations (GEE) model showing the effects of independent variables on mortality

Parameter	*B*	s.e.	95% Wald confidence interval		Hypothesis test		
			Lower	Upper	Wald, χ^2^	d.f.	*P*
(Intercept)	7.6	0.44	6.8	8.52	304.42	1	**<0.0001**
Female	0.28	0.12	0.04	0.52	5.40	1	**0**.**020**
Male	0^a^						
Ethnic group							
Black	0.36	0.1260	0.11	0.61	8.19	1	**0.004**
Asian	0.31	0.2512	−0.18	0.79	1.49	1	0.221
Mixed	0.77	0.6058	−0.41	1.96	1.628	1	0.202
Other	−0.04	0.34	−0.71	0.63	0.02	1	0.90
White	0^a^						
Age	−0.06^b^	0.0041	−0.07	−0.05	200.93	1	**<0**.**0001**
Health of the Nation Outcomes Scales	−0.07	0.0092	−0.09	−0.06	64.26	1	**<0**.**0001**
Number of discharges from acute hospitals	−0.01	0.0031	−0.02	−0.01	19.77	1	**<0**.**0001**
Index of Multiple Deprivation	0.01	0.0054	0.00	0.02	3.87	1	**0.049**
Modified Modified Continuity Index	−0.08	0.2675	−0.60	0.44	.09	1	0.754
Past or current smoker	0.19	0.1718	−0.15	0.52	1.22	1	0.268

Results in bold are significance.

a. This category is the reference category.

b. A − sign in front of the estimates (*B*) shows the direction of the relationship with the dependent variable. For example, the minus (−) in front of the *B* for age means that those surviving were younger.

This shows that those who did not die before final discharge were significantly more likely to be women, of Black ethnicity, younger, to have lower average total HoNOS scores, fewer admissions/discharges to non-psychiatric hospital and to live in less deprived areas. Continuity of care, smoking, and diagnosis did not have any significant independent effects on mortality in the model, and a history of comorbid substance misuse did not contribute at all.

Exploration of the contribution of individual HoNOS scale score to mortality is shown in Table 3.

**Table 3:** Contribution of demographic and individual Health of the Nation Outcomes Scales (HoNOS) scores to model

Parameter	*B*	s.e.	95% Wald confidence interval		Hypothesis test		
			Lower	Upper	Wald, χ^2^	d.f.	*P*
(Intercept)	6.79	0.47	5.868	7.721	206.744	1	**<0.001**
Female	0.26	0.12.4	0.014	0.499	4.292	1	**0.038**
Male	0^a^	–	–	–	–	–	–
Ethnic group							
Black	0.34	0.13	0.089	0.601	6.973	1	**0.008**
Asian	0.24	0.25	−0.255	0.744	0.919	1	0.338
Mixed	0.81	0.61	−0.405	2.024	1.706	1	0.191
Other	−0.21	0.34	−0.884	0.468	0.365	1	0.546
White	0^a^						
Past or current smoker	0.11	0.1887	−0.255	0.485	0.371	1	0.542
Age at start of year	−0.040	0.01	−0.049	−0.030	63.725	1	**<0.001**
Modified Modified Continuity Index for year	0.14	0.26	−0.370	0.660	0.303	1	0.582
Index of Multiple Deprivation for year	0.01	0.005	−0.001	0.021	2.959	1	0.085
Number of discharges from acute hospitals	−0.006	0.003	−0.012	−2.433 × 10^−5^	3.873	1	**0.049**
HoNOS scale 1 (overactive, aggressive, disruptive behaviour)	−0.15	0.076	−0.299	−0.001	3.877	1	**0.049**
HoNOS scale 3 (problems with drugs/alcohol)	−0.16	0.064	−0.291	−0.041	6.784	1	**0.009**
HoNOS scale 5 (problems with physical health)	−0.68	0.06	−0.807	−0.567	126.271	1	**<0.001**
HoNOS scale 6 (problems with hallucinations/delusions)	0.14	0.05	0.039	0.257	7.139	1	**0.008**
HoNOS scale 7 (problems with depressed mood) in year	−0.003	0.08	−0.168	0.162	0.001	1	0.974
HoNOS scale 8 (other mental and behavioural problems)	−0.028	0.06	−0.152	0.096	0.198	1	0.656
HoNOS scale 9 (problems with relationships)	0.192	0.065	0.064	0.320	8.596	1	**0.003**
HoNOS scale 10 (problems with ADLs)	−0.197	0.055	−0.307	−0.088	12.578	1	**<0.001**

Results in bold are significance.

ADLs, activities of daily living.

a. This category is the reference category.

This shows that of the six scales that contributed most to the overall total HoNOS association with mortality, the strongest positive associations were with scale 5 (physical health problems), scale 10 (activity of daily living problems) and scale 3 (problems with drugs/alcohol). Conversely, higher scores on scale 6 (problems with hallucinations/delusions) and scale 9 (problems with relationships) seemed to have a negative association with mortality.

### Causes of death deemed by ONS ‘treatable’ compared with other causes of death

There were 323 deaths of people over 74 (36.0%) so these were by definition excluded from the analysis of this ONS category. Of the remaining 575 patients who died, 90 (15.7%) died of ‘treatable’ causes according to ONS. These were compared with all who died in a GEE model. Lower age, higher total HoNOS and having an ICD-10 diagnosis of F20 versus F22 contributed independently to the model; continuity of care did not.

### Death because of self-injury

Suicide was the formal verdict in 17 patients. GEE analysis found no relationship between death by suicide and continuity of care. The circumstances of those with a formal suicide verdict and a further 23 who died from self-injury are shown in Table 4.

**Table 4:** Death by self-injury and coroner verdicts

Cause of death	Coroner verdict			Narrative	Open	Suicide	Total
	Accidental	Misadventure	Missing				
Drowned self	0	0	0	0	0	2	2
Fell from height	0	0	0	1	8	2	11
Hanged self	0	1	1	0	2	7	11
Head in plastic bag	0	0	0	0	0	1	1
Hit by train	0	0	0	3	0	0	3
Overdose illegal drugs	0	0	0	0	0	1	1
Overdose OTC medication	0	0	0	0	1	0	1
Overdose prescribed medication	0	0	0	0	1	1	2
Set fire to self or home	1	0	0	1	2	0	4
Stabbed self	0	0	1	0	0	1	2
Unknown	0	0	0	0	0	2	2
Total	1	1	2	5	14	17	40

OTC, over the counter.

The most parsimonious GEE model comparing these 40 patients with all other causes is presented in Table 5. For this analysis the total number of observations was 2791 and the total number of individuals analysed (without missing observations) was 724.

**Table 5:** Generalised estimating equations model comparing those dying from self-injury to all other causes of death

Parameter	*B*	s.e.	95% Wald confidence interval		Hypothesis test		
			Lower	Upper	Wald χ^2^	d.f.	*P*
(Intercept)	3.35	0.881	1.62	5.07	14.472	1	**<0.0001**
Main ICD diagnosis F20	−1.58	0.488	−2.53	−0.62	10.454	1	**0.001**
Main ICD diagnosis F22	0^a^						
Modified Modified Continuity Index	−0.19	0.091	−0.37	−0.02	4.658	1	**0.031**
Age	−0.09^b^	0.012	−0.12	−0.07	57.849	1	**<0.0001**
Not past or current smoker	−0.18	0.698	−1.55	1.18	0.069	1	0.793
Index of Multiple Deprivation	−0.01	0.007	−0.02	0.01	0.109	1	0.741
Health of the Nation Outcomes Scales Total	−0.01	0.005	−0.01	0.01	0.565	1	0.452

Results in bold are significance.

a. This category is the reference category.

b. The sign − in front of the estimates (B) shows the direction of the relationship with the dependent variable. For example the minus (−) in front of the B for age means that those who likely committed suicide were younger.

Those dying by self-injury were significantly younger, more often with a diagnosis of F22 compared with F20 and had lower relationship continuity of care. HoNOS score, IMD and smoking habits contributed to the final model but did not have significant independent effects. When only formal suicides versus all the other causes of deaths were analysed the only significant predictor was younger age (*β* = 0.102, Wald χ^2^ = 31.52, d.f. = 1, *P* < 0.001).

## Discussion

### Main findings

It does not seem that continuity of secondary mental healthcare has an independent relationship with overall mortality from all causes in schizophrenia, (or even those designated by ONS as having ‘treatable’ physical illness); thus we have not been able to confirm the results of Hoertel et al.^8^ We did find that lower relationship continuity of care in people with schizophrenia was independently related to deaths through self-injury compared with other deaths.

### Limitations

The study is subject to limitations. We were not able to assess confounding effects in the use of medication since this has hitherto been poorly captured by the electronic patient record. The results are from one single NHS trust and may not apply to others – the results are only possibly generalisable to continuity of secondary psychiatric care; as continuity in primary care may be more generally related to mortality in schizophrenia (other than by self-harm) as found by Hoertel et al.^8^ Unlike our GEE analysis, their survival analysis did not allow for correlations between continuity scores. Also, their study was of all psychiatric conditions and not in secondary care.

### Interpretation of our findings

We had expected higher continuity of secondary psychiatric care to be manifest in swifter recognition of physical illness and lifestyle problems and thus lower mortality. Although the HoNOS scale scores reflected these issues, it did not seem that continuity of care in psychiatric services had any effect on their final outcome. One possible explanation is that the management of comorbid physical conditions may actually be impaired by higher continuity: having different staff assessing the same patient may mean that physical disorders or risks that were not spotted by one staff member may be spotted by another. However, if present, this effect was not manifest in any inverse relationship in our data. Another explanation is that primary care relationship continuity is more important in reducing mortality than in secondary care.

In relation to deaths from all causes we found that younger age, lower HoNOS total scores, fewer non-psychiatric admissions to hospitals and Black ethnicity were independently associated with survival whereas male gender and higher deprivation were associated with mortality. Many of these factors are unsurprising. Separate HoNOS scale scores have been associated with mortality in a patient data-set overlapping with that in this study.^18^ The association of higher scores on HoNOS scale 5 (physical health problems) and scale 10 (activities of daily living problems) was to be expected,^18^ as was that with scale 3 (drugs/alcohol),^24^ although this was not apparent with a formal diagnosis of substance misuse.

It is not easy, however, to understand why higher scores on scale 6 (hallucinations and delusions) or scale 9 (relationship problems) should be independently associated with survival rather than death. One explanation is that these scales may reflect earlier stages in the evolution of schizophrenia, and thus an earlier stage in the evolution of the unknown factors that may eventually contribute to excess mortality, independent of age.

This effect was not apparent when only those with formal suicide verdicts were considered; suicide verdicts were given in less than half of these deaths. A criticism of this study is that the criteria for assigning death to self-injury were entirely clinical and based on case-note (chart) review by one investigator. However, given the increasing concern about the validity of formal suicide verdicts,^11,12^ this result suggests that new research methods to identify deaths because of self-injury in a more systematic and objective way are now required.

### Implications

It is well-known that patients with severe mental illness value relationship continuity with staff caring for them.^25,26^ It is not difficult to see how patients with psychosis who are suicidal might despair without stable long-term relationships with staff. It is also possible that lower continuity of care may contribute to less optimal treatment^27^ or psychosocial interventions.^28^ If evidence of declining clinical outcomes^13^ were not enough to stimulate a sustained effort to rectify the decline we have shown in continuity over recent years, will evidence of the consequent violent deaths of some of its most vulnerable patients?

Relationship continuity of care is at the heart of psychiatric care, if not all medical care. The apparent failure to protect this is mysterious to us. Perhaps what is needed is a resurrection of the clinical leadership from which the professions have too long, with much shrugging of shoulders, abdicated. If any further research is needed it might be to examine how this can be achieved.

## Data Availability

The data that support the findings of this study are not publicly available as they are subject to formal access restrictions in order to preserve the anonymity of patients. They can be made available on request from the corresponding author (A.M.), provided that the formal access criteria maintained by National Institute for Health Research, Office for National Statistics and the Hospital Episode Statistics systems are all met.

## Supplementary material

For supplementary material accompanying this paper visit http://dx.doi.org/10.1192/bjo.2021.965. click here to view supplementary material
